# Rayleigh-maximum-likelihood bilateral filter for ultrasound image enhancement

**DOI:** 10.1186/s12938-017-0336-9

**Published:** 2017-04-17

**Authors:** Haiyan Li, Jun Wu, Aimin Miao, Pengfei Yu, Jianhua Chen, Yufeng Zhang

**Affiliations:** School of Information Science and Engineering, Electronic Engineering, Yunnan University, No. 2 of North Cuihu Road, Kunming, 650091 China

**Keywords:** Ultrasound image enhancement, Noise, Speckle, Rayleigh-maximum-likelihood filter, Bilateral filter

## Abstract

**Background:**

Ultrasound imaging plays an important role in computer diagnosis since it is non-invasive and cost-effective. However, ultrasound images are inevitably contaminated by noise and speckle during acquisition. Noise and speckle directly impact the physician to interpret the images and decrease the accuracy in clinical diagnosis. Denoising method is an important component to enhance the quality of ultrasound images; however, several limitations discourage the results because current denoising methods can remove noise while ignoring the statistical characteristics of speckle and thus undermining the effectiveness of despeckling, or vice versa. In addition, most existing algorithms do not identify noise, speckle or edge before removing noise or speckle, and thus they reduce noise and speckle while blurring edge details. Therefore, it is a challenging issue for the traditional methods to effectively remove noise and speckle in ultrasound images while preserving edge details.

**Methods:**

To overcome the above-mentioned limitations, a novel method, called Rayleigh-maximum-likelihood switching bilateral filter (RSBF) is proposed to enhance ultrasound images by two steps: noise, speckle and edge detection followed by filtering. Firstly, a sorted quadrant median vector scheme is utilized to calculate the reference median in a filtering window in comparison with the central pixel to classify the target pixel as noise, speckle or noise-free. Subsequently, the noise is removed by a bilateral filter and the speckle is suppressed by a Rayleigh-maximum-likelihood filter while the noise-free pixels are kept unchanged. To quantitatively evaluate the performance of the proposed method, synthetic ultrasound images contaminated by speckle are simulated by using the speckle model that is subjected to Rayleigh distribution. Thereafter, the corrupted synthetic images are generated by the original image multiplied with the Rayleigh distributed speckle of various signal to noise ratio (SNR) levels and added with Gaussian distributed noise. Meanwhile clinical breast ultrasound images are used to visually evaluate the effectiveness of the method. To examine the performance, comparison tests between the proposed RSBF and six state-of-the-art methods for ultrasound speckle removal are performed on simulated ultrasound images with various noise and speckle levels.

**Results:**

The results of the proposed RSBF are satisfying since the Gaussian noise and the Rayleigh speckle are greatly suppressed. The proposed method can improve the SNRs of the enhanced images to nearly 15 and 13 dB compared with images corrupted by speckle as well as images contaminated by speckle and noise under various SNR levels, respectively. The RSBF is effective in enhancing edge while smoothing the speckle and noise in clinical ultrasound images. In the comparison experiments, the proposed method demonstrates its superiority in accuracy and robustness for denoising and edge preserving under various levels of noise and speckle in terms of visual quality as well as numeric metrics, such as peak signal to noise ratio, SNR and root mean squared error.

**Conclusions:**

The experimental results show that the proposed method is effective for removing the speckle and the background noise in ultrasound images. The main reason is that it performs a “detect and replace” two-step mechanism. The advantages of the proposed RBSF lie in two aspects. Firstly, each central pixel is classified as noise, speckle or noise-free texture according to the absolute difference between the target pixel and the reference median. Subsequently, the Rayleigh-maximum-likelihood filter and the bilateral filter are switched to eliminate speckle and noise, respectively, while the noise-free pixels are unaltered. Therefore, it is implemented with better accuracy and robustness than the traditional methods. Generally, these traits declare that the proposed RSBF would have significant clinical application.

## Background

Ultrasound imaging has been used as one of the most prevalent diagnostic techniques due to its advantage of being non-invasive, portable and cost-effective. However, ultrasound images are affected by many types of artifacts, therefore it is hard for an observer to interpret the images and obtain quantitative information from them. Noise in ultrasound can be modeled as the combined effect of two components: one is additive, such as electronic and thermal noise, and the other is multiplicative, called “speckle”. Speckle is the result of the constructive and destructive coherent summation of ultrasound echoes when ultrasound pulses randomly interfere with objects of comparable size to the sound wavelength and then the superposition of acoustical echoes produces an intricate interference pattern [1]. Noise and speckle, considered as undesirable consequence of the image formation process in coherent imaging, directly impact the visualization of the ultrasound image by the physician, deteriorate the quality and the perceivable resolution of diagnostically important features and thus lead to inaccuracy in clinical diagnosis. Therefore, it is essential to remove noise and speckle in the ultrasound images without compromising important image details.

**Fig. 1:**
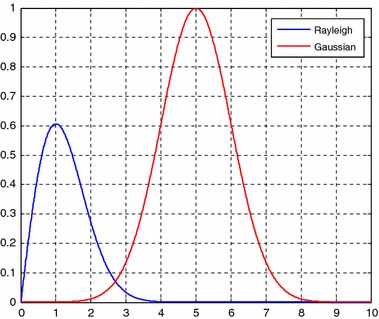
The Gaussian distribution of noise and the Rayleigh distribution of speckle

Prior to noise and speckle elimination, the major differences between noise and speckle in ultrasound image should be investigated. Besides the additive nature of noise and multiplicative nature of speckle, their statistical characteristics are completely different in that noise and speckle are subject to Gaussian distribution and Rayleigh distribution, respectively [1]. Figure 1 illustrates the Gaussian distribution whose mean is set as 5 and the variance is set as 1 as well as the Rayleigh distribution whose variance is set as 1. The difference should be considered for effective enhancement.

To alleviate the negative effects of noise and speckle, many efforts have been done to enhance ultrasound images and made the images more valuable after they had been generated and digitized. The state-of-the-art denoising methods for ultrasound images include the median filter [2], adaptive filters such as the Lee filter [3], the Frost filter [4], the Kuan filter [5] as well as the non-local means filter and the anisotropic diffusion method [6].

The median filtering [2] and numerous improved versions [7–9] are often effective for noise reduction. The median intensity of a properly sized and shaped filtering window surrounding the central pixel is used as the output of the target pixel. It thus can eliminate impulsive artifacts whose size is less than a half of the filtering window. Since the amount of smoothing performed by the median filter is determined only by the size of the filtering window, the median filter removes some of the high frequency signal while it results in obscuring the edges. Moreover, the median filter is ineffective when the speckle size is larger than a half of the window size since the filtering scheme does not take into account the statistical characteristics of speckle. Therefore, it undermines the despeckling effectiveness.

Statistical adaptive filters replace the regions within the image whose statistical characteristics similar to those of speckles with the local mean value while keeping the regions with property least similar to speckle unaltered. Therefore they remain effective for speckle suppression. Two of these works are the filters proposed by Lee [3] and Frost [4]. They were first applied on synthetic aperture radar images and then were used on ultrasound images. Lee used the minimum mean square error (MMSE) method to design the filter for additive noise, multiplicative noise and a mixed of the two, its output was estimated by a weighted average based on the mean and the variance of the sub-regions. The frost filter was designed by using an exponentially damped convolution kernel adapted to image fine details and the output was calculated based on local statistics. The Kuan filter [6] is closely similar to the Lee filter but with a different weighting function. Although these filters perform well for removing speckle, they have to compromise between averaging in homogeneous regions and preserving sharp features in the original image.

Non-local means (NLM), a weighted Gaussian filter was proposed for denoising by making use of the high degree of redundancy in the original ultrasound image [10]. NLM performs well in Gaussian noise suppression and sharp edge reservation since it uses the region comparison instead of pixel comparison, which the pattern redundancy is not restricted to be local. However, NL-means cannot be directly applied to ultrasound images since speckle differs from Gaussian noise significantly and is subject to Rayleigh distribution. To extend the application of the NLM method to speckle reduction, relevant NLM-based methods have been studied and proposed. Because these methods determine pixel similarity based on the noisy image patches, speckle in US images will lead to inaccurate computation of the similarity and thus lead to fine detail distortion [11–15]. Furthermore, additional time is required for computing the scale and shape parameters of the distribution of speckle [16].

The anisotropic diffusion was first proposed to reduce noise in images by smoothing in homogeneous regions without blurring the edges [17]. Thereafter, Yu and Acton [6] analyzed the statistical methods for speckle suppression and developed speckle reducing anisotropic diffusion (SRAD), a non-linear and space-variance filter. The SRAD approach reduced speckle by applying isotropic diffusion in homogeneous regions and enhanced edges by inhibiting diffusion across edges, which achieved a balance between despeckling and edge preservation. Flores et al. [18] extended the SRAD to a Log-Gabor guided anisotropic diffusion (ADLG), handling the trade-off between smoothing level and preservation of lesion contour details. Thereafter, a lot of work has been done with anisotropic diffusion equations in such a way that the important structural information can be retained in the denoised images [19–21]. But these SRAD based methods often produce a visually disappointing outputs when they are applied to filter the primary noise contained in ultrasound images, which is subject to Gaussian distribution.

Based on the assumption of Rayleigh distribution of speckle, Aysal [22] first proposed a Rayleigh-trimmed filter for speckle reduction in medical images. For ensuring high efficiency of removing the primary noise and speckle in ultrasound images, two filters are applied to the original image. An alpha-trimmed mean filter was used for suppressing the primary noise and the anisotropic diffusion was subsequently used to further reduce speckle. In addition, Deng et al. [23] proposed a Rayleigh-trimmed anisotropic diffusion filter for speckle reduction in ultrasound images. A Rayleigh-trimmed filter was first applied to estimate the relative standard deviations of local signals and then the anisotropic diffusion was utilized to reduce speckle. However, fine details were simultaneously removed by each filter because speckle detection was not performed before removing.

Elad proposed a new denoising method, named bilateral filter (BF) [24]. It is a non-linear weighted Gaussian filter, taking advantage of adaptive weights based upon spatial and radiometric similarity. Compared with the above-mentioned denoising methods, the BF replaced each pixel by a weighted average of the intensities in the window, which the weighting function gave high weight to those pixels near or similar to the central pixel. Hence it performed well in Gaussian noise reduction and sharp edges preservation. Lin [25] proposed a switching BF (SBF), where the BF could classify a pixel as Gaussian noise, impulse noise or noise-free one. And then the improved SBF switched between the Gaussian and impulse mode depending on the classification result to effectively remove Gaussian noise and impulse noise. However, these BF methods suffered from the drawback that they became ineffective when denoising speckle since the speckle model in ultrasonic images is subject to Rayleigh distribution.

Though these denoising and despeckling approaches operate well in some situations, they have several limitations. Firstly, some approaches do not take into account the statistical characteristics of noise or speckle, undermining the denoising effectiveness. Secondly, most algorithms, for example, the above mentioned approaches, except SBF, do not identify pixel property, such as noise, speckle or edge, before denoising, so they cannot balance effectively in enhancing edges and small structure while reducing noise and speckle, especially when the quality of the original image is poor. Finally, these methods can effectively suppress Gaussian noise or speckle but their performances are not satisfactory in the case of enhancing ultrasound images since ultrasound image is contaminated by addictive background noise and multiplicative speckle.

In order to effectively remove the primary noise and speckle contained in ultrasound images while preserving fine edges and details, a novel and robust method, named as Rayleigh-maximum-likelihood switching bilateral filter (RSBF), which performs the “detect and replace” mechanism before filtering, is proposed. To detect noise, a reference median [25] in the filtering window is first calculated based on the property of the edge in an image, and then the target pixel is identified as noise, speckle or noise-free texture according to the absolute difference between the target pixel and the reference median. Subsequently, noise is removed by the bilateral filter and speckle is suppressed by the Rayleigh-maximum-likelihood filter while noise-free pixels are kept unaltered. The performance and effectiveness of the proposed approach are demonstrated by experiments by using both simulated and clinical ultrasound images.

The remainder of this paper is organized as follows. "Methods" introduces the noise speckle model and the bilateral filter, followed by the detailed description of the proposed RSBF. "Experiments" gives a brief introduction to the experiment environment. "Results and discussion" presents the simulation of the ultrasound images and experiment results on synthetic ultrasound images and real clinical cases in which the proposed method is compared with six state-of-the-art methods for ultrasound speckle reduction. Finally, some conclusions are drawn in "Conclusions".

## Methods

In this section, the characteristics of noise and speckle is first analyzed. Based on the analysis, a sorted quadrant median vector (SQMV) scheme is performed to classify the target pixel as noise, speckle or noise-free. Thereafter, the bilateral filter is applied to remove noise based on the Gaussian distribution of noise. And the Rayleigh-maximum-likelihood filter is proposed to suppress speckle based on the Rayleigh distribution of speckle. Noise free pixels are kept unchanged in order to enhance images and meanwhile preserve the edge details.

### The ultrasound noise model and the bilateral filter

#### Thermal noise and speckle model

Artifacts in ultrasound images contain additive electronic or thermal noise and multiplicative speckle. When an image is corrupted by thermal noise, each original pixel value is added with a noise value $n_{i,j}$ produced from a zero-mean Gaussian distribution. Then the noisy image $u_{i,j}$ is related to the original image $f_{i,j}$ by:

1 $$ u_{i,j} = f_{i,j} + n_{i,j} $$

Speckle is an interfering phenomenon. It occurs when two or more waves traveling to the probe from the scatters interfere with each other, whose severity depends on the relative phase between two overlapping returning ultrasonic echoes. A reasonable trade-off between accuracy and simplicity is to model the speckle as a multiplicative artifact with Rayleigh distribution [26]. The relationship between the uncorrupted signal $f_{i,j}$ and the observed signal $u_{i,j}$ contaminated by speckle is:

2 $$ u_{i,j} = f_{i,j} *\eta_{i,j} $$

where $\eta$ is a Rayleigh probability density function with the scale parameter $\sigma$:

3 $$ p(\eta ) = \frac{\eta }{{\sigma^{2} }}e^{{\left( { - \frac{{\eta^{2} }}{{2\sigma^{2} }}} \right)}} $$

The noisy ultrasound image, $u_{i,j}$ contaminated by noise and speckle can be modeled as:

4 $$ u_{i,j} = n_{i,j} + f{}_{i,j} * \eta_{i,j} $$

#### The bilateral filter

The Bilateral filter was proposed to remove Gaussian noise while preserving edges [24]. Each pixel is replaced by a weighted average of the intensities in the filtering window, where the pixels near or similar to the target one are assigned a high weight.

In a $(2N + 1)*(2N + 1)$ window, let $u_{i,j}$ and $u_{i + s,j + t}$ represent the value of the central pixel and the pixels surrounding the central one, respectively, where $(i,j)$ and $(i + s,j + t)$ indicate the location of $u_{i,j}$ and $u_{i + s,j + t}$. Then the filtered result of the bilateral filter $y_{i,j}$ is defined as:

5 $$ y_{i,j} = \frac{{\sum\nolimits_{s = - N}^{N} {\sum\nolimits_{t = - N}^{N} {W_{G} (s,t)W_{R} (s,t)u_{i + s,j + t} } } }}{{\sum\nolimits_{s = - N}^{N} {\sum\nolimits_{t = - N}^{N} {W_{G} (s,t)W_{R} (s,t)} } }} $$

where

6 $$ W_{G} (s,t) = \exp - \frac{{(i - s)^{2} + (j - t)^{2} }}{{2\sigma_{S}^{2} }} $$

7 $$ W_{R} (s,t) = \exp - \frac{{(u_{i,j} - u_{i + s,j + t} )^{2} }}{{2\sigma_{R}^{2} }} $$

$\sigma_{S}$ and $\sigma_{R}$ are the two parameters that control the bilateral filter, whose values are determined based on experiments.

The bilateral filter performs well in suppressing Gaussian noise while keeping the edge, but it is hard to remove ultrasound speckle because speckle is a type of multiplicative noise and it follows Rayleigh distribution. In order to effectively filter noise and speckle in ultrasound image, we propose a Rayleigh-maximum-likelihood bilateral filter (RSBF) with noise/speckle detection scheme, discussed in the following.

### The Rayleigh-maximum-likelihood switching bilateral filter(RSBF)

**Fig. 2:**
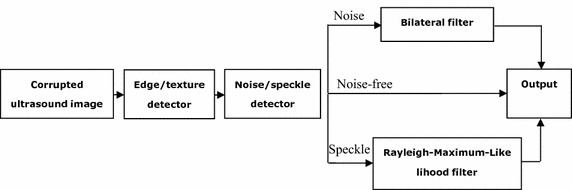
The scheme of noise/speckle detection and reduction by RSBF

The Rayleigh-maximum-likelihood bilateral filter (RSBF) consists of two steps. Firstly, the target pixel is identified as noise, speckle or noise-free by using a sorted quadrant median vector [25]. Subsequently, noise and speckle are suppressed by the bilateral filter and the Rayleigh-maximum-likelihood filter, respectively, while noise-free pixels are left unaltered. Figure 2 shows the coarse structure of the proposed method.

#### Noise/texture detection with the sorted quadrant median vector [25]

1. The Sorted Quadrant Median Vector (SQMV)

**Fig. 3:**
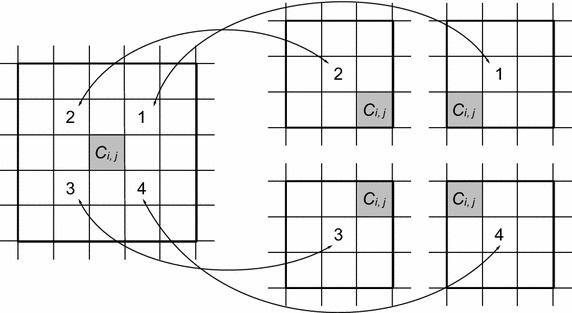
Four subwindows in a $5 \times 5$ window

SQMV was proposed for noise detection to avoid false noise detection or blurring image details with inappropriate window size [25]. A large window with the size of $(2N + 1) \times (2N + 1)$ is divided into four subwindows of size $(N + 1) \times (N + 1)$, where the central pixel is the corner pixel in the four subwindows, shown in Fig. 3 ($N = 2$).

Let $C_{i,j}$ be the intensity of the central pixel in the large window, then the set of pixels within the window, denoted as $W$, is defined as:

8 $$ W = \{ C_{i + x,j + y} : - N \le x \le N, - N \le y \le N\} $$

The set of pixels in the four subwindows, denoted as $W_{1} ,W_{2} ,W_{3}$ and $W_{4}$, can be expressed as

9 $$ W_{1} = \{ C_{i + x,j + y} :0 \le x \le N,0 \le y \le N\} $$

10 $$ W_{2} = \{ C_{i + x,j + y} : - N \le x \le 0,0 \le y \le N\} $$

11 $$ W_{3} = \{ C_{i + x,j + y} : - N \le x \le 0, - N \le y \le 0\} $$

12 $$ W_{4} = \{ C_{i + x,j + y} :0 \le x \le N, - N \le y \le 0\} $$

In case that $N = 2$ then the window size is $5 \times 5$ and there are four sub-windows with size of $3 \times 3$. The sequence of the sub-windows is shown in Fig. 3. The median value of each sub-window is denoted as:

13 $$ m_{j} = median\{ W_{j} \} ,\quad j = 1\;to\;4 $$

The $SQMV$ is defined as the four median values in the subwindows in a sorted increasing order:

14 $$ SQMV = [SQM_{1} ,SQM_{2} ,SQM_{3} ,SQM_{4} ] $$

where $SQM_{i} (i = 1,2,3,4)$ is the median of the four subwindows sorted in an increasing order.

2. Edge/texture detection based on the $SQMV$ clusters

The edge and texture pixels in a window can be detected based on the difference between the maximal and the minimal $SQMV$ values:

15 $$ SQM_{\text{max} } = (SQM_{4} - SQM_{1} ) $$

16 $$ SQM_{\text{min} } = (SQM_{3} - SQM_{2} ) $$

where $SQM_{\text{max} }$ and $SQM_{\text{min} }$ represent the maximal and the minimal value of the $SQMV$, respectively.

If $SQM_{\text{max} }$ is small, then the pixel intensities in the window are close to each other, so there is no edge. In case that $SQM_{\text{max} }$ is large but $SQM_{\hbox{min} }$ is small, then the window contains a weak edge. If $SQM_{\hbox{max} }$ and $SQM_{\hbox{min} }$ are both large, then the pixel intensities are different, so the target pixel is identified as strong edge or texture. Based on the analysis, the edge and texture can be detected by:

17 $$ Edge /texture = \left\{ {\begin{array}{llll} {without \,edge} &  & {SQM_{\text{max} } \le T} \\ {weak\, edge} & \hfill & {SQM_{\text{max} } \ge T} \hfill & {\& \, SQM_{\text{min} } \le T} \hfill \\ {strong\, edge/texture} & \hfill & {SQM_{\text{min} } \ge T} \hfill  \\ \end{array} } \right. $$

where T is a threshold.

3. Features of edge/texture

The cluster of $SQM_{i}$ and the order of four medians $m_{j}$ mapping to the clusters implicate the edge features in the window. (1) If $SQM_{i}$ is clustered as two unequal classes, such as $\{ (SQM_{1} ,SQM_{2} ,SQM_{3} ,),(SQM_{4} )\}$ or $\{ (SQM_{1} )(SQM_{2} ,SQM_{3} ,SQM_{4} )\}$ then a diagonal edge is contained in the window. (2) If $SQM_{i}$ is clustered as two equal classes, $\{ (SQM_{1} ,SQM_{2} )(SQM_{3} SQM_{4} )\}$, furthermore, $\{ (m_{2} ,m_{3} )\}$ and $\{ (m_{1} ,m_{4} )\}$ are associated to $\{ SQM_{1} ,SQM_{2} \}$ and $\{ SQM_{3} ,SQM_{4} \}$, respectively, then there is a vertical edge within the window. (3) If $SQM_{i}$ is clustered as two equal classes,$\{ (SQM_{1} ,SQM_{2} )(SQM_{3} SQM_{4} )\}$, furthermore, $\{ (m_{1} ,m_{2} )\}$ and $\{ (m_{3} ,m_{4} )\}$ are associated to $\{ (SQM_{1} ,SQM_{2} )\}$ and $\{ (SQM_{3} ,SQM_{4} )\}$, respectively, then the window contains a horizontal edge.

4. Reference median

**Fig. 4:**
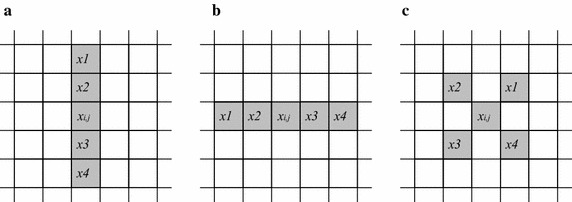
The four pixels in the major patter of **a** vertical, **b** horizontal, **c** diagonal directions

If “without edge” or “weak edge” are detected by Eq. (17), the majority feature of the window is denoted by the medians in the major cluster. Thus the reference median is defined as the average of $SQM_{2}$ and $SQM_{3}$. When there is edge or texture in the window, a directional average, used to determine which subwindow the target pixel is more similar to, is calculated by the average of the four pixels in the major pattern depending on the feature of edge or texture, expressed by $ref$.

18 $$ ref = \left\{ {\begin{array}{*{20}l} {(SQM_{2} + SQM_{3} )/2} \hfill & {without\, edge\quad \& \;weak\, edge} \hfill \\ {\left( {\sum\limits_{i = 1}^{4} {x_{i} } } \right)/4} \hfill & {strong\, edge /texture} \hfill \\ \end{array} } \right. $$

where $x_{i} (i = 1,2,3,4)$ are the four pixels in the major pattern, defined according to the feature of edge or texture, shown in Fig. 4.

If $ref$ is close to $(SQM_{1} ,SQM_{2} )$ then the reference median is defined as $SQM_{2}$. If $ref$ is close to $(SQM_{3} ,SQM_{4} )$ then the reference median is defined as $SQM_{3}$. The reference median ($SQMR$) can be expressed as:

19 $$ SQMR\begin{array}{*{20}c} = & {\left\{ {\begin{array}{*{20}l} {(SQM_{2} + SQM_{3} ) /2} \hfill & {SQM_{\text{min} } \le T} \hfill &{} \hfill \\ {SQM_{2} } \hfill & {SQM_{\text{min} } \ge T} \hfill &{\& \, ref \in (SQM_{1} ,SQM_{2} )} \hfill \\ {SQM_{3} } \hfill & {SQM_{\text{min} } \ge T} \hfill & {\& \, ref \in (SQM_{3} ,SQM_{4} )} \hfill \\ \end{array} } \right.} \\ \end{array} $$

5. Noise and edge/texture identification

The noise, speckle and edge/texture detection mechanism is implemented by the reference median and the threshold $T$. When the target pixel is greatly different from the reference median then it is detected as speckle generated by ultrasound echoes [1]. When the target pixel is close to the reference median then it may be a Gaussian background noise or edge/texture. Then the noise and edge/texture is further identified based on the clusters of $SQMV$ by using Eq. (17). The noise/speckle and edge/texture detection algorithm is shown as follows:

20 $$ noise /speckle\;detector = \left\{ {\begin{array}{lll} {speckle} \hfill & {\left| {x_{i,j} - SQMR} \right| \ge T_{1} } \hfill \\ {noise} \hfill & {\left| {x_{i,j} - SQMR} \right| \ge T_{2} } \hfill \\ {noise{ - }free} \hfill  &\quad{otherwise} \hfill \\ \end{array} } \right. $$

where $T_{1}$ and $T_{2}$ are thresholds determined by experiments.

#### The Rayleigh-maximum-likelihood filter

Speckle in ultrasound images is modeled as a multiplicative form defined by Eq. (2). The estimation of uncorrupted image $f$ can be adopted by the robust maximum likelihood (ML) approach [22]. $f(i,j)$ is assumed to be a constant in the observation window $\varOmega$ from which $f(i,j)$ is to be estimated [22], i.e., $f(i,j) \approx \beta$ for $\forall (i,j) \in \varOmega$. Hence, for $\{ u(i,j):(i,j) \in \varOmega \}$ defined in Eq. (2) can be calculated by using:

21 $$ u(i,j) = \beta *\eta (i,j) $$

where $(i,j)$ denotes the coordinates of a pixel in the ultrasound image. $u$, denoting the image corrupted by speckle, is a Rayleigh distribution with parameter $\sigma_{I}$. The ML estimation of $\sigma_{I}$, represented as $\hat{\sigma }$, is given by

22 $$ \hat{\sigma }_{I} = \mathop {\arg \hbox{max} }\limits_{\beta } \psi (\vec{u}|\sigma_{I} ) $$

where $\vec{u}$ denotes the pixel values of $u(i,j)$ in the observation window $\varOmega$ arranged in a vector format. The solution to Eq. (22) is

23 $$ \hat{\sigma }_{I} = \left( {\frac{1}{2\left\| \varOmega \right\|}\sum\limits_{(i,j) \in \varOmega } {u^{2} (i,j)} } \right)^{1/2} $$

where $\left\| \varOmega \right\|$ denotes the cardinality of $\varOmega$. For example, $\left\| \varOmega \right\|$ = 9 in a $3 \times 3$ window. Then the ML estimation of the uncorrupted image $f(i,j)$, denoted as $\hat{f}(i,j)$, can be calculated as

24 $$ \begin{aligned} \hat{f}(i,j) & = \hat{\beta } = \hat{\sigma }_{I} (\sigma )^{ - 1} \\ & = (\sigma )^{ - 1} \left[ {\frac{1}{2\left\| \varOmega \right\|}\sum\limits_{(i,j) \in \varOmega } {u^{2} (i,j)} } \right]^{1/2} \\ \end{aligned} $$

where $(i,j)$ denotes the pixel location of the estimated original image.

#### The Rayleigh-maximum-likelihood bilateral filter

Inspired by the “noise/speckle detection and reduction” scheme and statistics of noise and speckle, we combine the bilateral filter and Rayleigh-maximum-likelihood filter together after performing noise/texture identification. Firstly, the target pixel is classified as noise, speckle or noise-free one. Subsequently, noise is removed by using the bilateral filter and speckle is suppressed by using the Rayleigh-maximum-likelihood filter while the noise free pixels are kept unaltered. The detailed algorithm is described below, which can be directly implemented by MATLAB or C language.

For each pixel $u_{i,j}$ in the noisy image:

1. Take the search window, size of $(2N + 1) \times (2N + 1)$, and the corresponding subwindows size of $(N + 1) \times (N + 1)$.
2. Compute $SQMV$ by Eq. (14) and detect the edge and texture by using Eq. (17).
3. Identify edge feature according to the cluster of $SQMV$.
4. Calculate $ref$ by Eq. (18) and $SQMR$ by Eq. (19).
5. Detect $u_{i,j}$ as noise, speckle or noise-free pixel.
6. If $u_{i,j}$ is classified as noise, replace the target pixel with a weighted average intensities in the window by using Eq. (5).
7. If $u_{i,j}$ is detected as speckle, filter the target pixel with the Rayleigh-maximum-likelihood filter in the window by using Eq. (24).
8. If $u_{i,j}$ is noise-free then the original intensity is kept unaltered.

## Experiments

In the experimental study, synthetic and clinical ultrasound images are used as test sources to evaluate the performance of the proposed RSBF by comparing it with those of six previously proposed filters, including the switching bilateral filter (SBF), the median filter, the SRAD, the NL-means filter, the Lee filter and the Kuan filter.

The proposed RSBF is implemented by using Matlab 7.1 and the experiments are performed on a PC with 2.66 GHz Intel Core 2 processor. Here the parameter $\sigma_{S}$ and $\sigma_{R}$ of the bilateral filter are set as 35 and 40 based on the experiments, respectively. The parameter $\sigma$ of the Rayleigh-maximum-likelihood filter is set as 1. The size of the observation window is $5 \times 5$.

Three widely used quantitative metrics, including signal to noise ratio (SNR) [26], peak signal to noise ratio (PSNR) [27] and root mean squared error (RMSE) [28], are used to measure the effectiveness of various algorithms along with the visual evaluation. The SNR is defined as

25 $$ {\text{SNR\_dB}} = 10\log \left( {\frac{{\sum\nolimits_{i = 1,j = 1}^{i = Row,j = Col} {f(i,j)^{2} } }}{{\sum\nolimits_{i = 1}^{row} {\sum\nolimits_{j = 1}^{col} {[\hat{f}(i,j) - f(i,j)]^{2} } } }}} \right) $$

where $f(i, j)$ is the original image, $\hat{f}(i,j)$ is the denoised image, and the size of image is $Row \times col$. The PSNR reflects the average statistics of the signal-to-noise ratio change in an image, and is defined as

26 $$ PSNR\_dB = 10\log \left( {\frac{{255^{2} \times {\text{Row}} \times Col}}{{\sum\nolimits_{i = 1}^{Row} {\sum\nolimits_{j = 1}^{Col} {[f(i,j) - \hat{f}(i,j)]^{2} } } }}} \right) $$

The higher value of SNR and PSNR indicate better performance of denoising.

The RMSE is defined as

27 $$ RMSE = \sqrt {\frac{{\left( {\sum\nolimits_{i = 1}^{row} {\sum\nolimits_{j = 1}^{col} {(f(i,j) - \hat{f}(i,j))^{2} } } } \right)}}{Row \times Col}} $$

The less the RMSE, the better the image quality.

## Results and discussion

### Synthetic images with speckle

**Fig. 5:**
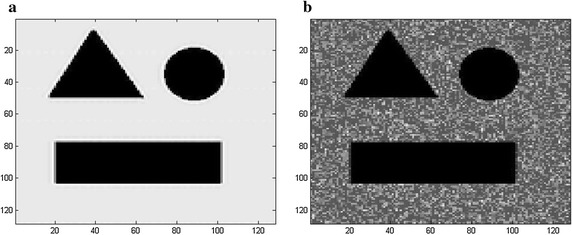
Speckle simulation **a** the original image. **b** The noisy image (SNR = 6 dB)

In this paper, synthetic ultrasound images contaminated by speckle are simulated by using the speckle model proposed by Laporte [29] and the details of this simulation model can be found in the work by Laporte et al. [29]. The corrupted synthetic images are generated by the original image multiplied with Rayleigh distributed noise of various SNR levels. A simulated image with speckle generated with this model (SNR = 6 dB) is shown in Fig. 5.

To visually evaluate the performance of the proposed method compared with other methods, seven algorithms, including the proposed method, the SBF, the Median filter, the SRAD, the NL-means filter, the Lee filter and the Kuan filter, are applied to the simulated images. The windows size of the Median filter and the Lee filter are $5 \times 5$, the iteration time of the SRAD is 300.

**Fig. 6:**
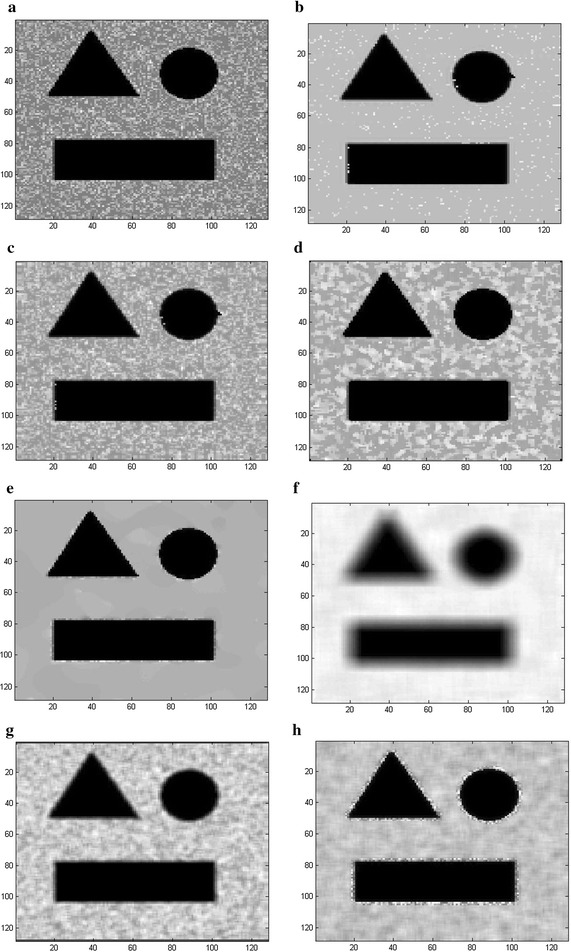
The visual experiment results for **a** the synthetic image corrupted with speckle (SNR = 12 dB), the output by **b** the proposed RSBF, **c** SBF filter, **d** Median filter, **e** SRAD, **f** NL-means, **g** Lee filter and **h** Kuan filter

**Fig. 7:**
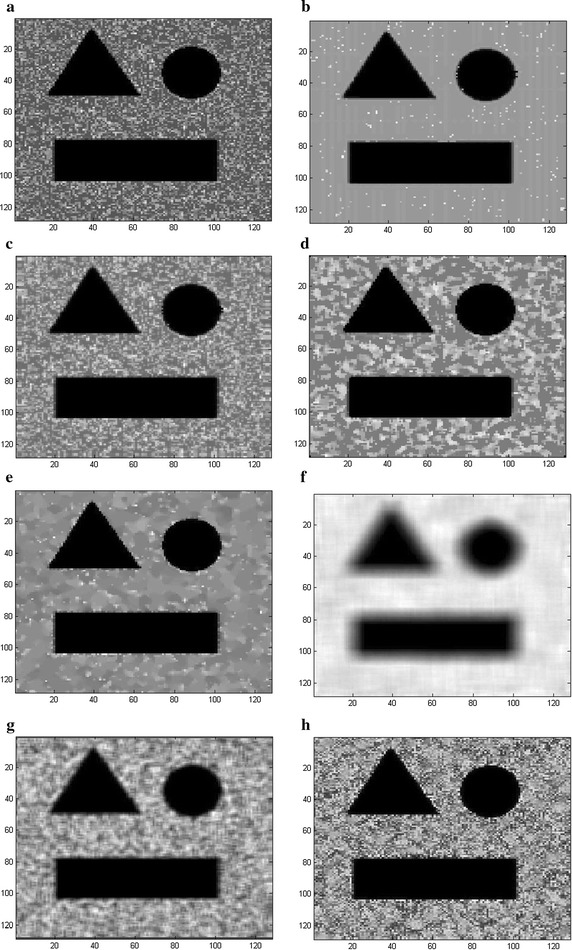
The visual experiment results for **a** the synthetic image corrupted with speckle (SNR = 5 dB), the output by **b** the proposed RSBF, **c** SBF filter, **d** Median filter, **e** SRAD, **f** NL-means, **g** Lee filter and **h** Kuan filter

Figures 6 and 7 illustrate the results of the seven filters at SNR = 12 dB (shown in Fig. 5a) and SNR = 5 dB (shown in Fig. 6a), respectively. Figures 6b–h and 7b–h show the corresponding filtered outputs of the proposed method, SBF, Median, SRAD, NL-means, Lee and Kuan, respectively. The visual quality of Fig. 6e is not as good as that of Fig. 6b, since the result obtained by the proposed method (Fig. 6b) contains sharp boundaries but no artifacts. The SRAD (Fig. 6e) can suppress the speckle but it meanwhile blurs the edges. However, the SBF (Fig. 6c), the Median (Fig. 6d), the NL-means (Fig. 6f), the Lee (Fig. 6g) and Kuan filter (Fig. 6h) can hardly remove the Rayleigh distributed speckle, and a mass of speckle is clearly distributed in the background. The performance of the SRAD greatly degrades when the image quality is poor and obvious speckle appears in the background (Fig. 7e), while the proposed method (Fig. 7b) can achieve the result which is more similar to the ground truth and filter the speckle more effectively.

The quantitative effectiveness of the proposed method is confirmed by the indices of the SNR, PSNR and RMSE, as shown in Tables 1, 2 and 3, respectively. The SNR of the corrupted images are 1, 3, 5, 7, 10, 12, 15 dB. The SRAD can obtain better results than those by the proposed method when the image quality is high (SNR = 15 dB), whereas at low SNRs (SNR = 7, 5, 3 and 1 dB), the performance of SRAD decreases significantly. It can hardly eliminate the speckle. But the proposed method still holds high SNR and PSRN as well as low RMSE values, outperforming SRAD. In all cases, the achievements of the proposed methods are better than those of the SBF, Median, NL-means, Lee and Kuan filters. It is worth noting that the proposed method can improve the SNRs of the filtered images to nearly 15 dB compared with the corrupted image under various SNR levels. It can thus be concluded that the proposed RSBF can reduce noise and speckle effectively while simultaneously keeping edges and fine details.

**Table 1:** The SNR comparison of the synthetic images corrupted by speckle under different noise levels (dB)

Noise image	SNR = 15 dB	SNR = 12 dB	SNR = 10 dB	SNR = 7 dB	SNR = 5 dB	SNR = 3 dB	SNR = 1 dB
Proposed	30.5639	28.2034	25.8091	23.9633	21.6541	18.3528	16.2720
SBF	22.3715	19.0805	16.9216	13.8543	11.9803	9.9208	8.0415
Median	19.7635	17.1132	15.2007	12.3096	10.4227	8.1360	5.6368
SRAD	31.5266	26.3032	19.3339	11.5845	8.1085	5.0146	2.6095
NL-means	19.6304	19.5233	19.3788	18.9897	18.7536	18.1153	16.0211
Lee	20.3398	18.8931	17.5508	15.3167	14.0998	12.1313	10.306
Kuan	24.9467	18.9903	17.7373	15.7109	14.0892	12.0872	10.3109

**Table 2:** The PSNR comparison of the synthetic images corrupted by speckle under different noise levels (dB)

Noise image	SNR = 15 dB	SNR = 12 dB	SNR = 10 dB	SNR = 7 dB	SNR = 5 dB	SNR = 3 dB	SNR = 1 dB
Proposed	40.0128	40.1669	39.9244	37.6524	35.2580	33.4122	31.1031
SBF	31.4337	28.1467	25.9877	22.9204	21.0456	18.9869	17.0807
Median	28.8297	26.1794	24.2668	21.3757	19.4888	17.2021	14.7029
SRAD	40.5927	35.3693	28.4001	20.6506	17.1747	14.0807	11.6757
NL-means	28.6967	28.5895	28.4449	28.0558	27.8197	27.1815	25.5873
Lee	35.0228	33.1100	31.9598	30.6620	30.0365	29.6552	29.0178
Kuan	29.3561	28.0564	26.8034	24.7770	23.1643	21.1534	19.3770

**Table 3:** The RMSE comparison of the synthetic images corrupted by speckle under different noise levels

Noise image	SNR = 15 dB	SNR = 12 dB	SNR = 10 dB	SNR = 7 dB	SNR = 5 dB	SNR = 3 dB	SNR = 1 dB
Proposed	0.00008	0.00017	0.00013	0.00014	0.00032	0.00058	0.00074
SBF	0.0007	0.0015	0.0023	0.0072	0.0071	0.0115	0.0182
Median	0.0013	0.0024	0.0036	0.0072	0.0104	0.0177	0.0306
SRAD	0.00013	0.00037	0.0014	0.0083	0.0174	0.0355	0.0624
NL-means	0.0018	0.0019	0.0019	0.0020	0.0021	0.0024	0.0031
Lee	0.0014	0.0018	0.0022	0.0029	0.0041	0.0056	0.0096
Kuan	0.0012	0.0016	0.0021	0.0033	0.0048	0.0077	0.0115

### The synthetic images corrupted by speckle and Gaussian noise

In this section, the synthetic image is generated by multiplying the original image with Rayleigh-distributed speckle of various levels, and then additive Gaussian noise of zero mean with the variance of 0.002 is mixed into obtain the noisy images.

**Fig. 8:**
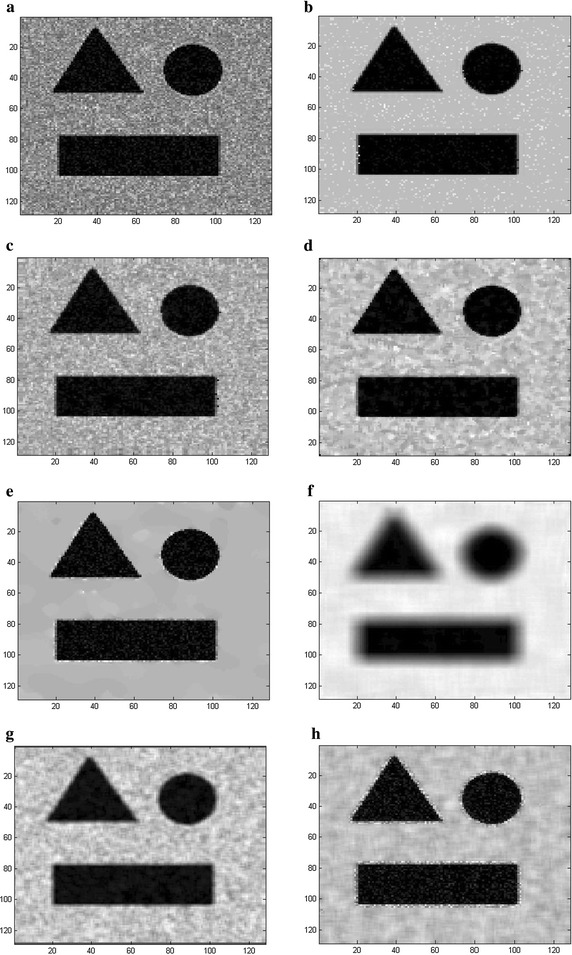
The visual experiment results for **a** the synthetic image corrupted with speckle and Gaussian noise (the variance of Gaussian is 0.002, SNR = 12 dB), the output by **b** the proposed RSBF, **c** SBF filter, **d** Median filter, **e** SRAD, **f** NL-means, **g** Lee filter and **h** Kuan filter

**Fig. 9:**
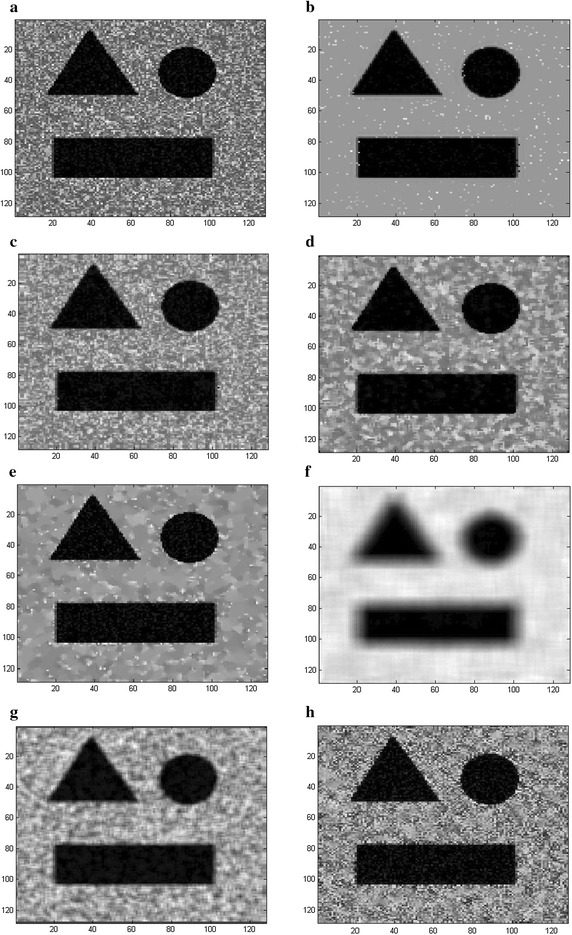
The visual experiment results for **a** the synthetic image corrupted with speckle and Gaussian noise (the variance of Gaussian is 0.002, SNR = 5 dB), the output by **b** the proposed RSBF, **c** SBF filter, **d** Median filter, **e** SRAD, **f** NL-means, **g** Lee filter and **h** Kuan filter

Figures 8 and 9 show the visual performance of the proposed method in comparison with the SBF, Median, SRAD, NL-means, Lee and Kuan filters at SNR = 12 dB (shown in Fig. 8a) and SNR = 5 dB (shown in Fig. 9a), respectively. It can be observed that the SRAD (Fig. 8e) suppresses the speckle significantly but it distorts the edges of ROI (region of interest) and the output inevitably contains background noise even when the image quality is high (SNR = 12 dB). However, the SBF (Fig. 8c), the Median (Fig. 8d), the NL-means (Fig. 8f), the Lee (Fig. 8g) and Kuan filter (Fig. 8h) cannot effectively remove the speckle and background noise, whose outputs include considerable distortion and illegibility. The SRAD cannot correctly eliminate the speckle or Gaussian background noise and the image is greatly deteriorated (Fig. 9e), leaving a lot of residual speckle and noise in the recovered results. Figures 8 and 9 show that the proposed method (Figs. 8b, 9b) can perform better for edge preserving as well as speckle and noise suppression than other methods do since the proposed method identify a pixel as speckle, noise or noise-free prior to removing, and it subsequently remove noise and speckle by the bilateral filter and the Rayleigh-maximum-likelihood filter, respectively, while the noise-free pixel is kept unchanged.

Table 4 shows that our method has a larger SNR index than those of the other methods. Compared with the SRAD, the index values obtained by the proposed RSBF are 14 dB higher than those obtained by SRAD when the SNRs of the noisy image are not greater than 7 dB. Therefore, it can be concluded that our method produces images with more structural similarity with the noiseless image than other methods do and our method is more effective and robust than the compared approaches, especially when the image quality is poor. This uniformity can be confirmed by the quantitative indices of the PSRN and RMSE, as shown in Tables 5 and 6.

**Table 4:** The SNR comparison of the synthetic images corrupted by speckle and Gaussian noise under different noise levels (dB)

Noise image	SNR = 15 dB	SNR = 12 dB	SNR = 10 dB	SNR = 7 dB	SNR = 5 dB	SNR = 3 dB	SNR = 1 dB
Proposed	27.7353	28.3570	28.3151	25.0501	21.7660	19.7310	17.5150
SBF	21.9157	18.7967	16.6018	13.7071	11.8087	9.9209	7.8395
Median	20.7309	17.6163	15.4225	11.3534	10.2713	8.0520	5.6002
SRAD	25.5620	22.8780	17.9199	11.3920	7.9691	5.1211	2.5685
NL-means	17.8788	17.7779	17.7091	17.4288	17.1081	16.8585	15.1291
LEE	18.5462	18.2778	16.7909	15.1985	13.4685	12.1603	10.1315
Kuan	23.5905	21.8682	19.6651	13.0058	8.8894	5.7629	3.2307

**Table 5:** The PSNR comparison of the synthetic images corrupted by speckle and Gaussian noise under different noise levels (dB)

Noise image	SNR = 15 dB	SNR = 12 dB	SNR = 10 dB	SNR = 7 dB	SNR = 5 dB	SNR = 3 dB	SNR = 1 dB
Proposed	37.1843	37.8060	37.7641	34.3361	31.7660	29.1620	26.9639
SBF	31.3647	28.2465	26.0508	23.1560	21.2576	19.3698	17.2884
Median	30.1799	27.0653	24.8715	20.8024	19.7203	17.5010	15.0511
SRAD	35.0140	32.3199	27.3698	20.8410	17.4187	14.5701	12.0175
NL-means	27.3287	27.2269	27.1580	26.8778	26.5577	26.3074	24.5780
Lee	34.4946	32.7927	31.6582	30.5488	30.0358	26.7189	24.7268
Kuan	36.1296	34.8856	33.1143	29.7408	28.5548	26.1472	24.8663

**Table 6:** The RMSE comparison of the synthetic images corrupted by speckle and Gaussian noise under different noise levels

Noise image	SNR = 15 dB	SNR = 12 dB	SNR = 10 dB	SNR = 7 dB	SNR = 5 dB	SNR = 3 dB	SNR = 1 dB
Proposed	0.00019	0.00016	0.00017	0.00037	0.00076	0.0012	0.0020
SBF	0.00073	0.0015	0.0025	0.0060	0.0075	0.0116	0.0187
Median	0.00096	0.0020	0.0033	0.0083	0.0107	0.0178	0.0313
SRAD	0.00032	0.00059	0.0018	0.0082	0.0181	0.0349	0.0628
NL-means	0.0019	0.0020	0.0021	0.0022	0.0023	0.0027	0.0035
Lee	0.0015	0.0021	0.0027	0.0036	0.0058	0.0092	0.0121
Kuan	0.0013	0.0016	0.0025	0.0045	0.0072	0.0103	0.0236

### Clinical imaging data

**Fig. 10:**
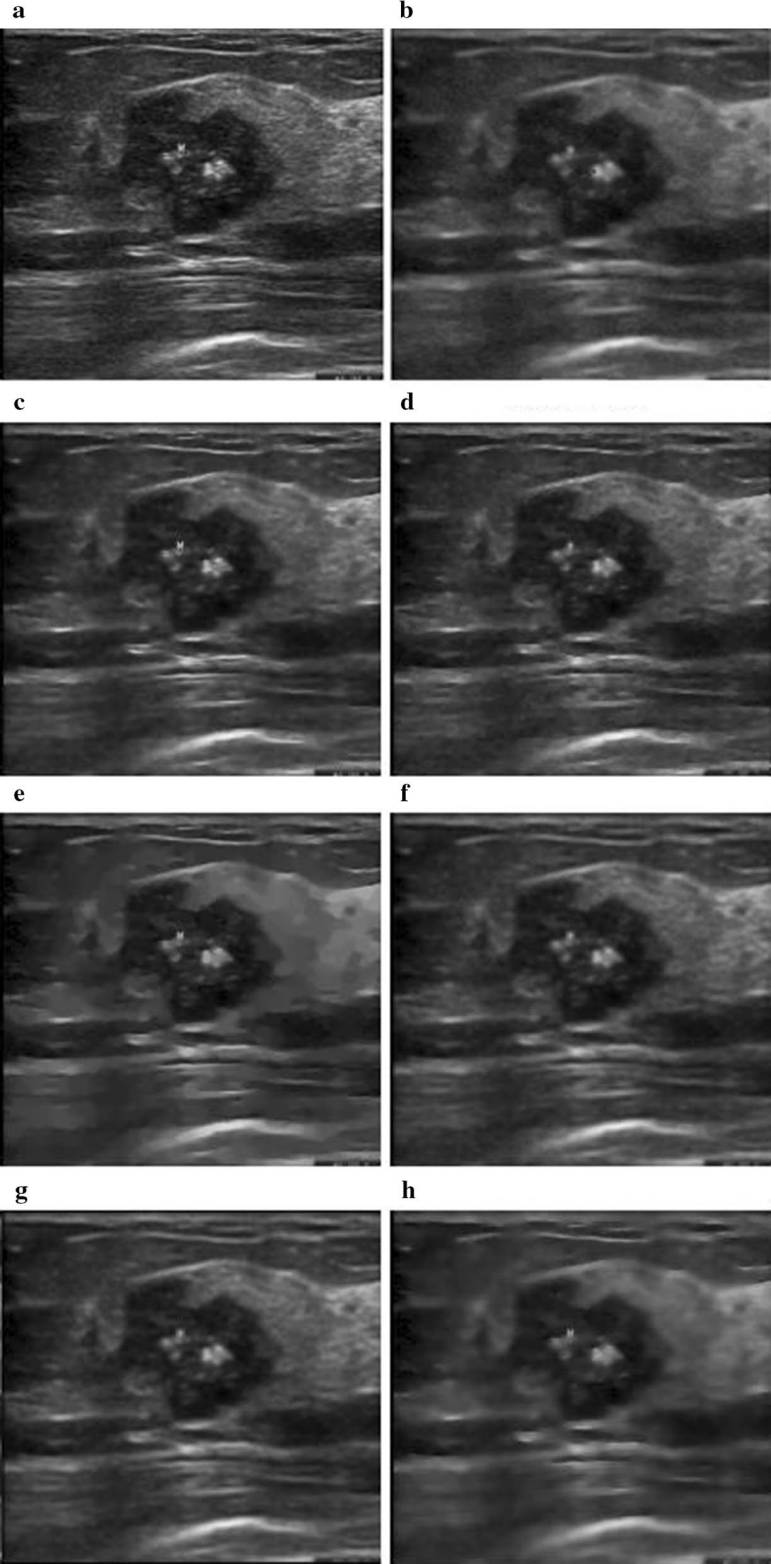
The visual experiment results for **a** the clinic breast ultrasound image, the output by **b** the proposed RSBF, **c** SBF filter, **d** Median filter, **e** SRAD, **f** NL-means, **g** Lee filter and **h** Kuan filter

Experiments are also performed on one dataset of real ultrasound breast images. The clinical breast (Fig. 10a) ultrasound images are collected by using Philips Envisor 2540A ultrasound system, provided by the third hospital affiliated to the Kunming medical university, and are used with patients’ consent.

Figure 10a is the original image selected from the experimental dataset. Figure 10b–h are the results obtained by the proposed method, the SBF, the Median, the SRAD, the NL-means, the Lee and the Kuan filters, respectively. It can be seen that the pixel intensity of the ROI processed by the proposed method is smoother than those of six other methods, which demonstrates that our method is more effective in enhancing edge while smoothing the speckle and noise in clinical ultrasound image.

## Conclusions

A novel and robust method, RSBF, has been proposed to remove speckle and background noise in ultrasound images by implementing a “detect and replace” two-step mechanism. Firstly, each central pixel in the observation window is classified as noise, speckle or noise-free texture according to the absolute difference between the target pixel and the reference median, which is calculated based on the property of the edge in an image. Subsequently, a Rayleigh-maximum-likelihood filter and a bilateral filter are switched to eliminate speckle, assumed to be Rayleigh distributed, and noise, subjecting to Gaussian distribution, respectively, while keeping the noise-free pixel unaltered. Experiments are performed on synthetic and clinical ultrasound images by comparing seven different despeckling methods. Visual evaluation and three numerical indices are applied to evaluate the performance of the proposed method. Results show that the proposed method performs effectively in speckle and noise suppression as well as edge preservation, and is superior to some well-accepted state-of-the-art filters in despeckling, especially when the image quality is poor.

The first conclusion is that the proposed method yields excellent noise/speckle attenuation and edge enhancement because it detects the target pixel as speckle, noise and noise-free before performing filter. Besides, our method achieves robust performance at various image quality levels, especially when the image is greatly deteriorated because the switched filters take into account the statistics of speckle and noise.

In the application of the RSBF, the parameter $\sigma_{S}$ and $\sigma_{R}$ of the bilateral filter and the parameter $\sigma$ of the Rayleigh-maximum-likelihood filter are set as fixed. It is not adaptive in processing the real clinical ultrasound images. Thus, how to optimize the parameters needs to be further researched.

In addition, even though the proposed method is theoretically suitable for ultrasound images of various organs, such as abdominal ultrasound images, the experiment only performs on one dataset of breast ultrasound images due to the limitation of the data. Therefore, more clinic ultrasound images of various organs should be utilized to test the performance of the proposed method in future research.

## Abbreviations

RSBF: Rayleigh-maximum-likelihood switching bilateral filter
SQMV: sorted quadrant median vector
SNR: signal to noise ratio
PSNR: peak signal to noise ratio
RMSE: root mean squared error
